# Acute kidney injury in COVID-19 patients receiving remdesivir: A systematic review and meta-analysis of randomized clinical trials

**DOI:** 10.1016/j.clinsp.2023.100200

**Published:** 2023-04-13

**Authors:** Golnaz Shams, Asma Kazemi, Khatereh Jafaryan, Mohammad Hossein Morowvat, Payam Peymani, Iman Karimzadeh

**Affiliations:** a Pharmaceutical Sciences Research Center, Shiraz University of Medical Sciences, Shiraz, Iran; b Nutrition Research Center, Shiraz University of Medical Sciences, Shiraz, Iran; c Department of Clinical Pharmacy and Pharmacy Practice, School of Pharmacy and Pharmaceutical Sciences, Isfahan University of Medical Sciences, Isfahan, Iran; d College of Pharmacy, University of Manitoba, Winnipeg, Manitoba, Canada; e Department of Clinical Pharmacy, School of Pharmacy, Shiraz University of Medical Sciences, Shiraz, Iran

**Keywords:** SARS-COV-2, COVID-19, Acute Kidney Injury, Remdesivir, Systematic review, Meta-analysis

## Abstract

**Objectives:**

Remdesivir is an antiviral agent with positive effects on the prognosis of Coronavirus Disease (COVID-19). However, there are concerns about the detrimental effects of remdesivir on kidney function which might consequently lead to Acute Kidney Injury (AKI). In this study, we aim to determine whether remdesivir use in COVID-19 patients increases the risk of AKI.

**Methods:**

PubMed, Scopus, Web of Science, the Cochrane Central Register of Controlled Trials, medRxiv, and bioRxiv were systematically searched until July 2022, to find Randomized Clinical Trials (RCT) that evaluated remdesivir for its effect on COVID-19 and provided information on AKI events. A random-effects model meta-analysis was conducted and the certainty of evidence was evaluated using the Grading of Recommendations Assessment, Development, and Evaluation. The primary outcomes were AKI as a Serious Adverse Event (SAE) and combined serious and non-serious Adverse Events (AE) due to AKI.

**Results:**

This study included 5 RCTs involving 3095 patients. Remdesivir treatment was not associated with a significant change in the risk of AKI classified as SAE (Risk Ratio [RR]: 0.71, 95% Confidence Interval [95% CI] 0.43‒1.18, p = 0.19, low-certainty evidence) and AKI classified as any grade AEs (RR = 0.83, 95% CI 0.52‒1.33, p = 0.44, low-certainty evidence), compared to the control group.

**Conclusion:**

Our study suggested that remdesivir treatment probably has little or no effect on the risk of AKI in COVID-19 patients.

## Introduction

The Coronavirus Disease (COVID-19) has brought about 600 million infections and around 6 million deaths globally. Over the last two years, many drugs have been studied for efficacy against Severe Acute Respiratory Syndrome Coronavirus-2 (SARS-CoV-2) infection.^1^, ^2^, ^3^, ^4^ Remdesivir is an antiviral drug whose clinical trials started soon after the emergence of COVID-19, which resulted in obtaining emergency use authorization from the US Food and Drug Administration (FDA) in May 2020, and also from several other countries later. The pandemic is under better control since the vaccination roll-out began. In addition, new antivirals like nirmatrelvir/ritonavir and molnupiravir have been developed to curb the death toll of the COVID-19 pandemic.^5^^,^^6^ However, the virus is still circulating and remdesivir is yet the drug of choice specifically in countries that have no access to the newly approved drugs. The remdesivir formulations contain sulfobutylether-beta-cyclodextrin, an excipient that accumulates in patients with renal dysfunction.^7^ In the remdesivir trials, patients with impaired kidney function were excluded and it was generally suggested that renal function should be monitored in all the patients receiving the treatment. Therefore, concerns regarding the kidney safety profile of remdesivir were raised because even a minor acute reduction in kidney function could have important clinical consequences for patients. A study using international pharmacovigilance post-marketing databases (VigiBase), detected a significant signal of nephrotoxicity associated with remdesivir.^8^ Two other pharmacovigilance studies that analyzed post-marketing reports in FDA Adverse Events Reporting System (FAERS), suggested Acute Kidney Injury (AKI) as the most frequent Adverse Event (AE) following treatment with remdesivir^9^ and reported that AKI in COVID-19 patients was significantly associated with remdesivir use.^10^ However, a meta-analysis of two Randomized Clinical Trials (RCT) showed that remdesivir has little or no effect on the risk of AKI.^11^ Since then, the results of other randomized trials of remdesivir have been published. Therefore, a systematic review and meta-analysis was conducted to address the question of whether treating COVID-19 patients with remdesivir results in an alteration in the risk of AKI, compared to the patients taking the Standard Of Care (SOC) or placebo.

## Methods

This systematic review and meta-analysis were performed according to the Preferred Reporting Items for Systematic Reviews and Meta-Analyses (PRISMA) statement.^12^ The protocol was registered on the International Prospective Register of Systematic Reviews (PROSPERO: CRD42022313410).

### Inclusion and exclusion criteria

All RCTs on patients of any age with laboratory-confirmed or clinically suspected COVID-19 with the intervention included remdesivir alone or in combination with SOC compared with a placebo or the SOC without remdesivir were included. Studies in which different doses of remdesivir were compared if at least 2 trials compared the same doses were also considered eligible for inclusion. The trials were included regardless of publication status and with no restrictions on language. Studies were excluded if they did not report on the AKI events, or if the participants were pregnant.

The primary outcomes were AKI classified as a Serious Adverse Event (SAE) and the combined serious and non-serious AEs due to AKI defined as the number of patients with the event.

### Literature search

A thorough systematic search of databases including PubMed, Scopus, Web of Science, the Cochrane Central Register of Controlled Trials, medRxiv, and bioRxiv was conducted with no restrictions on language. In addition, the trial records submitted to ClinicalTrials.gov and the WHO International Clinical Trials Registry Platform were also searched. The initial search was performed on 1 May 2022 and rerun on 30 July 2022. The detailed search strategy is shown in Table S1.

### Data extraction

Titles and abstracts were assessed for eligibility by two independent reviewers (G.S, K.J). Full-text articles of the selected studies were assessed by the same two reviewers (G.S, K.J). If the reviewers were unable to reach a consensus, they consulted a third review author (I.K). Data were extracted by two investigators (G.S and K.J), independently. Any discrepancies between the review authors were resolved through discussion with a third reviewer (A.K). The following data were retrieved from each eligible study: trial registration, first author, the abbreviation of each trial, year of publication, region of trial, study design, number of patients, participant characteristics (age, sex, comorbidities), remdesivir dose and duration, control intervention, the proportion of patients with different levels of severity of COVID-19 at baseline, data source of the outcome, and outcomes of interest. The severity of the disease in the included trials was defined based on different scales. Thus, the authors reported the severity of the disease based on the proportion of patients receiving respiratory support at baseline as follows: no oxygen, low-flow oxygen, and mechanical ventilation (including non-invasive mechanical ventilation, high-flow oxygen, and invasive mechanical ventilation).^13^

### Risk of bias assessment

The revised version of the Cochrane tool for assessing the risk of bias in RCTs (RoB 2.0) was used to evaluate the risk of bias for the included studies and each outcome.^14^ Two reviewers assessed the risk of bias independently (G.S, A.K) using the RoB 2.0 Excel tool to manage and record assessments. RoB 2.0 comprises five domains: bias arising from the randomization process; bias due to deviations from the intended interventions; bias due to missing outcome data; bias in the measurement of the outcome; and bias in the selection of the reported result. Each domain was categorized as “low risk of bias”, “some concerns”, or “high risk of bias”.

### Certainty of the evidence assessment

Certainty of the evidence was assessed according to the Grading of Recommendations Assessment, Development, and Evaluation (GRADE) process, based on the five domains including the risk of bias, inconsistency, indirectness, imprecision, and publication bias, rated as very low, low, moderate, and high.

### Statistical analysis

Data retrieved from the included studies were recorded into a Microsoft Excel spreadsheet. Analyses were performed using Stata software version 13 (StataCorp LP, College Station, TX, USA). The risk Ratio (RR) and corresponding 95% Confidence Interval (95% CI) were calculated to obtain the effect of the intervention on the primary and secondary outcomes and pooled using the DerSimonian and Laird method which takes into account the between-study variation.^15^ The Cochran's Q test and I^2^ values were calculated for measuring the amount of heterogeneity. Sensitivity analyses were performed by excluding one study at a time and reestimating the effect sizes to make sure that the results are not influenced by one large study or a study with an extreme result.

## Results

### Trials selection and characteristics

**Fig. 1:**
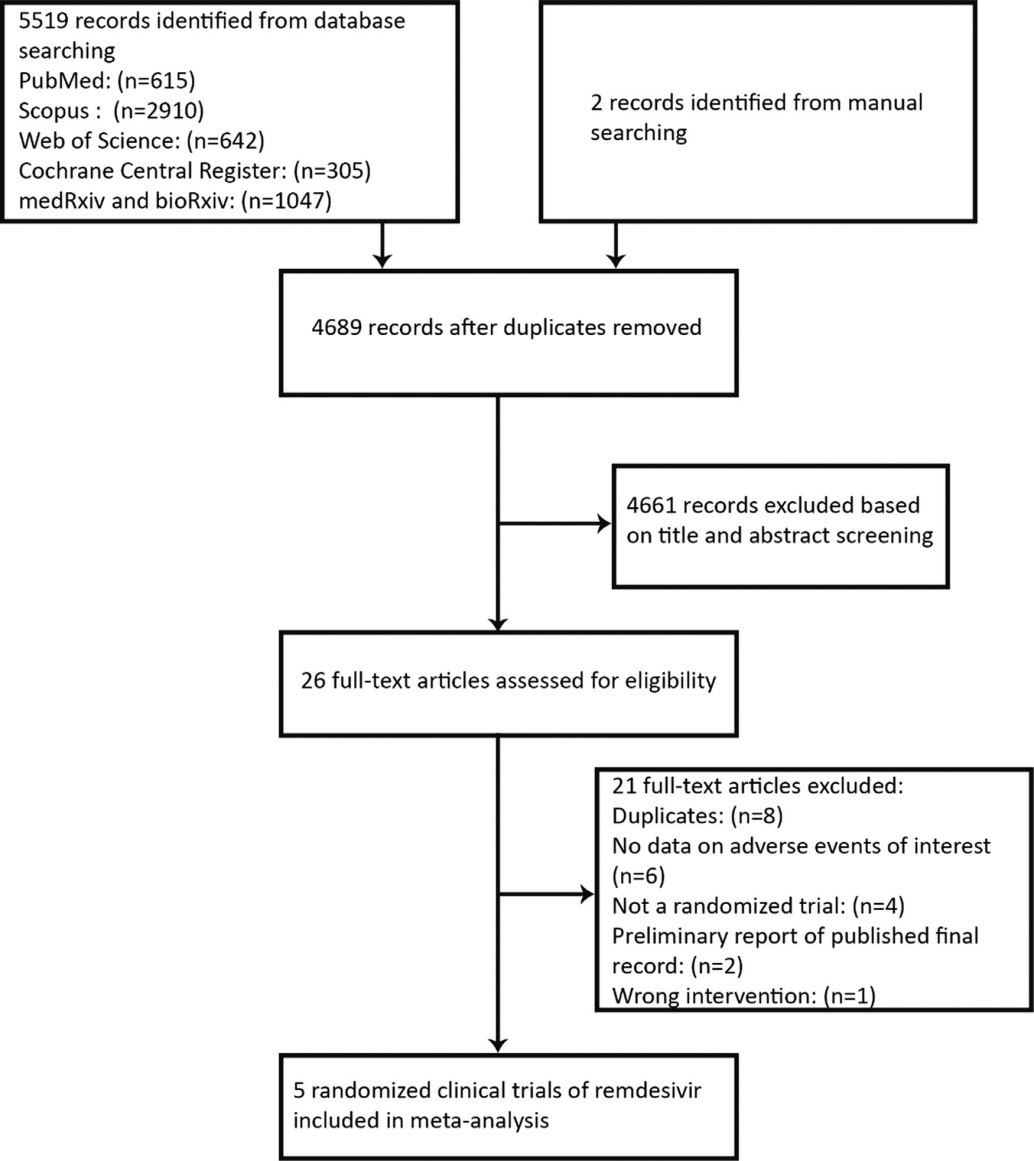
Flow diagram of identification, screening, inclusion, and exclusion of trials.

Initially, 5519 records were retrieved from databases and 2 others by manual searching. After removing 832 duplicates, screening through titles and abstracts excluded 4661 records and 26 articles left for full-text screening. Among them, 11 published RCTs for remdesivir were found and 15 records were further excluded. The WHO Solidarity trial is the largest published RCT of remdesivir.^16^ However, assessing the adverse effects was not among the outcomes of this study, so this trial was excluded from the meta-analysis. Three add-on studies to the WHO Solidarity trial led by Canada,^17^ France,^18^^,^^19^ and Norway^20^ recorded other outcomes and reported their results independently. Among them, only the study conducted by France named the DisCoVeRy trial reported the number of AEs due to AKI. The final results of the DisCoVeRy trial which was available as a preprint was used for the present meta-analysis.^19^ Other RCTs were also excluded for not reporting the outcome of interest.^21^, ^22^, ^23^ Finally, 5 RCTs were included in this meta-analysis. The selection flow diagram is shown in Fig. 1.

Table 1 shows the major characteristics of included studies. The study by Wang et al. was a randomized, double-blind, placebo-controlled, multicenter trial (NCT 04257656) of 237 COVID-19 patients (age ≥ 18 years) admitted to the hospital. Patients who received continuous renal replacement therapy or those with an estimated Glomerular Filtration Rate (eGFR) < 30 mL/min were not included in this trial. The study had 2 arms (remdesivir for 10 days vs. placebo for 10 days). This study was terminated early due to the control of the outbreak in Wuhan, China.^24^ The Adaptive COVID-19 Treatment Trial (ACTT-1) was a double-blind, randomized, placebo-controlled, multicenter trial by Beigel et al. (NCT 04280705), consisting of 1062 COVID-19 patients (age ≥18 years) admitted to the hospital. Patients with eGFR < 30 mL/min or those receiving hemofiltration or hemodialysis were excluded from entering the trial. This study compared remdesivir for 10 days with a placebo for the same duration.^25^ The SIMPLE study in moderate COVID-19 patients by Spinner et al. (NCT 04292730), was a randomized, open-label, multicenter trial of 596 hospitalized patients (age ≥12 years) comparing 5- or 10-days remdesivir with the SOC. The exclusion criteria for this study was eGFR < 50 mL/min at baseline .^26^ DisCoVeRy was a randomized, open-label, adaptive, multicenter, controlled trial by Ader et al. (NCT 04315948). 1308 hospitalized COVID-19 patients (age ≥18 years) were randomized to receive SOC alone or in combination with remdesivir for 10-days, lopinavir-ritonavir, lopinavir-ritonavir plus interferon beta-1a, or hydroxychloroquine. In this study, the arms containing lopinavir-ritonavir or hydroxychloroquine stopped prematurely,^27^ and the results for remdesivir were published separately.^18^^,^^19^ Patients with eGFR < 30 mL/min or those under dialysis, were not included in the trial. The SIMPLE study in severe COVID-19 patients by Goldman et al. was a randomized, open-label trial (NCT 04292899) of 397 patients (age ≥ 12 years), comparing 5-day and 10-day remdesivir regimens. Participants with baseline eGFR < 50 mL/min were excluded from this trial.^28^

**Table 1:** Characteristics of the included trials.

Study	Study design and setting	Country	Male (%)	Age (median/mean) (years)	Comparator	Dose and duration of remdesivir	Severity at baseline (%)			Comorbidities (%)	Data source of outcome	Follow-up (days)
							No oxygen	Low flow	High flow/ ventilated			
Ader 2022 DisCoVeRy NCT04315948	Open-label, adaptive, multicenter, inpatient	France, Belgium, Austria, Portugal, Luxembourg	69.5	64	Standard care (n = 428)	200 mg IV on Day 1, 100 mg on Days 2‒10 (n = 429)	1.8	57.2	41.0	Obesity (34.1), Chronic cardiac disease (27.9), Diabetes mellitus (26.8), Chronic pulmonary disease (18.0), Smoking (current or former) (17.6), Chronic kidney disease (6.6)	Publication, Preprint	90
Beigel 2020 ACTT-1 NCT04280705	Double-blind, multicenter, inpatient	United States, Denmark, United Kingdom, Greece, Germany, Korea, Mexico, Spain, Japan, and Singapore	64.4	58.9	Placebo (n = 521)	200 mg IV on Day 1, 100 mg on Days 2‒10 (n = 541)	13.0	41.0	45.0	Hypertension (51), Obesity (46), Diabetes (31), Asthma (12), coronary artery disease (13), Neoplasm malignant (8), Chronic kidney disease (7), Chronic respiratory disease (7)	Publication, Clinicaltrials.gov	28
Spinner 2020 SIMPLE-Moderate NCT04292730	Open-label, multicenter, inpatient	United States, China, France, Germany, Hong Kong, Italy, Japan, Korea, Netherlands, Singapore, Spain, Sweden, Switzerland, Taiwan, United Kingdom	61.1	NR	Standard care (n = 200)	200 mg IV on Day 1, 100 mg on Days 2‒5 (n = 199) or 200 mg IV on Day 1, 100 mg on Days 2‒10 (n = 197)	84.1	15.0	0.9	Cardiovascular disease (56.3), Hypertension (42.5), Diabetes (39.7), Asthma (13.9)	Publication, Clinicaltrials.gov, Clinical Study Report	28
Wang 2020 NCT04257656	Double-blind, multicenter, inpatient	China	59.3	65	Placebo (n = 79)	200 mg IV on Day 1, 100 mg on Days 2‒10 (n = 158)	1.3	82.2	16.5	Hypertension (43.2), Diabetes (23.7), Coronary heart disease (7.2)	Publication	28
Goldman 2020 SIMPLE-Severe NCT04292899	Open-label, multicenter, inpatient	United States, Italy, Spain, Germany, Hong Kong, Singapore, South Korea, Taiwan	63.7	NR	‒	200 mg IV on Day 1, 100 mg on Days 2‒5 (n = 200) or 200 mg IV on Day 1, 100 mg on Days 2‒10 (n = 197)	13.9	55.4	30.7	Hypertension (49.9), Diabetes (22.7), Hyperlipidemia (22.4), Asthma (12.3)	Publication, Clinicaltrials.gov	28

N, Number; IV, Intravenous; ACTT-1, Adaptive COVID-19 Treatment Trial-1; NR, Not Reported.

### Risk of bias and certainty of evidence assessment

**Fig. 2:**
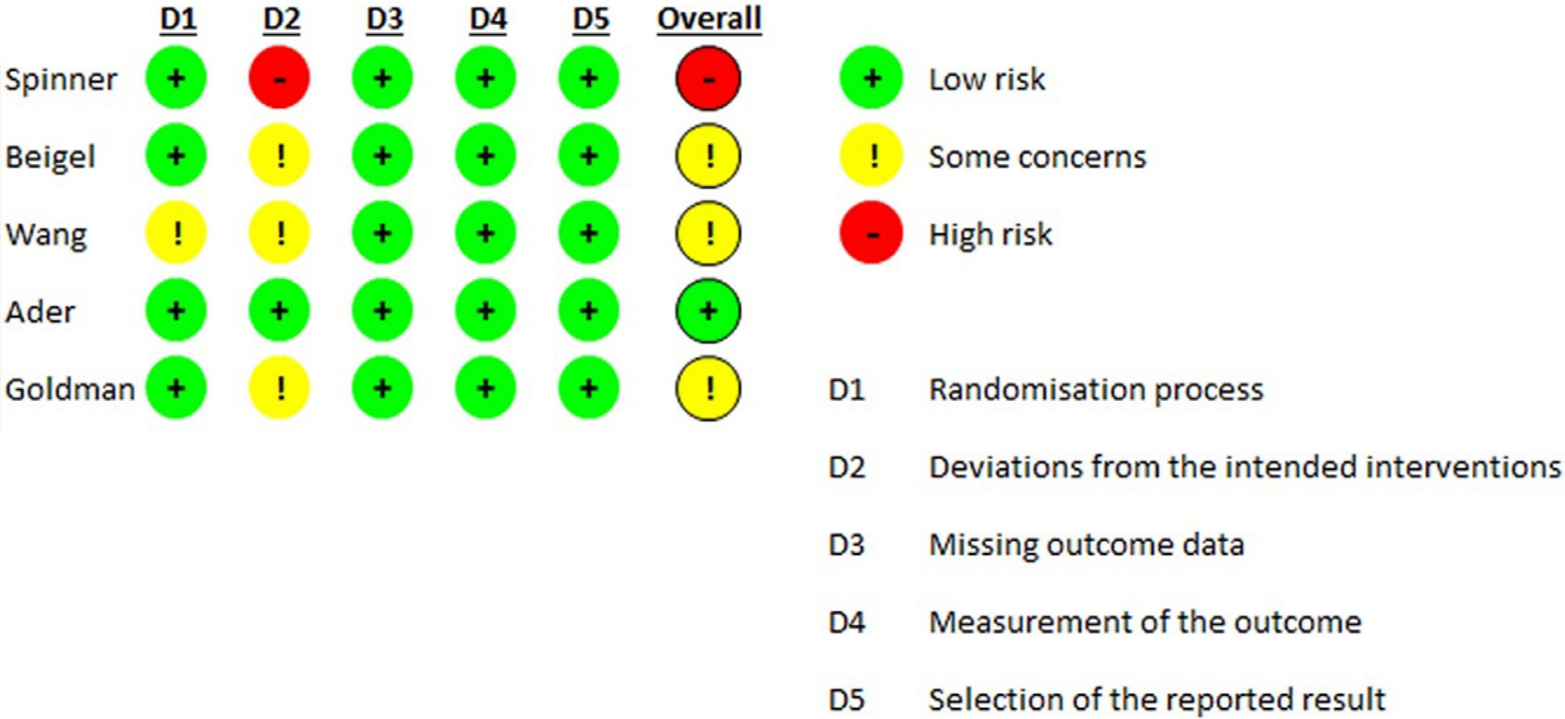
Summary of risk of bias in included studies.

The risk of bias assessment is summarized in Fig. 2 and Fig. S1. The Ader study has a low risk of bias. The studies by Beigel, Wang, and Goldman were judged with some concerns, due to the inappropriate analyses. The study by Wang also has some baseline variations between the placebo and remdesivir groups. The Spinner study was judged with a high risk of bias because the percentage of the patients treated with concomitant medications is significantly higher in the SOC groups and is not balanced between study arms. The open-label design of this study might have led to this imbalance which is likely to affect the number of adverse events. The certainty of the evidence, assessed through the GRADE methodology was low for SAEs and any grade AE due to AKI and was downgraded to very low certainty for the effect of the 10-day vs. 5-day remdesivir course of treatment on AKI (Table S2).

### AKI classified as SAE

**Fig. 3:**
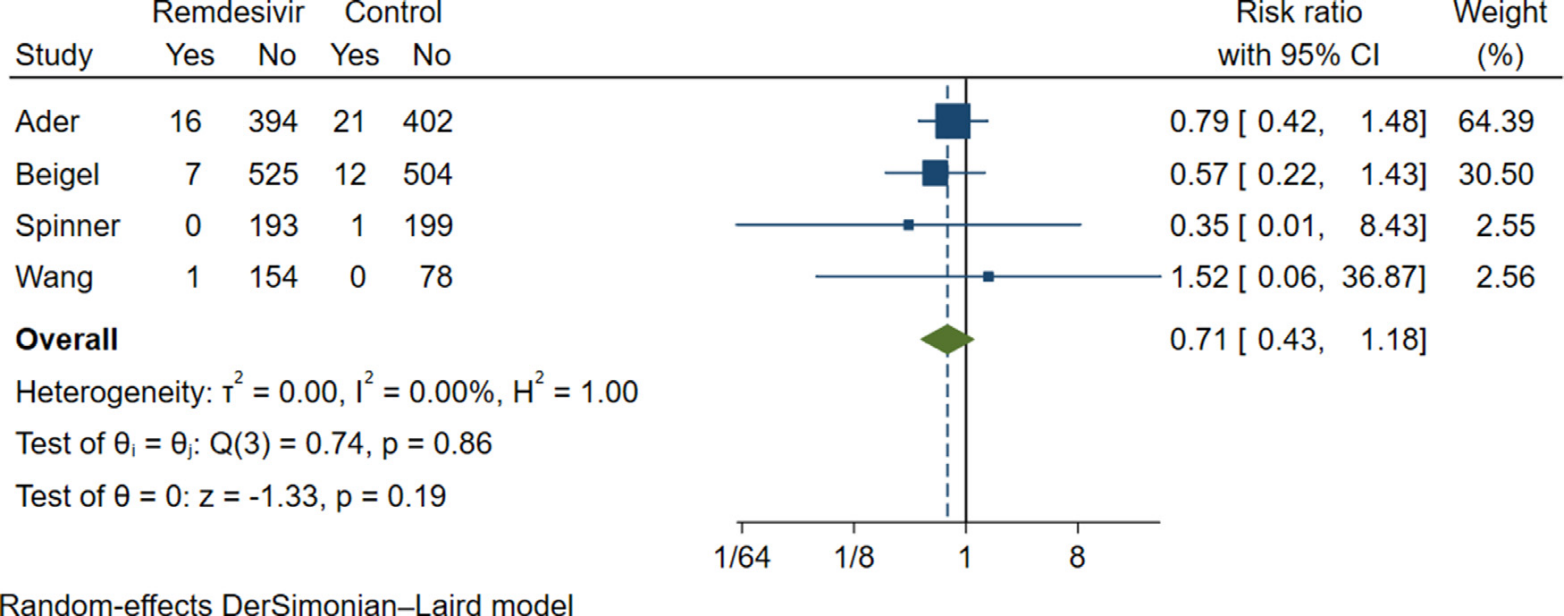
Forrest plot comparing the effect of remdesivir vs. control on AKI events classified as serious adverse events.

In 4 RCTs comparing remdesivir with the control group, with a total of 2507 hospitalized COVID-19 patients, AKI was reported as SAEs. In 1290 patients who received remdesivir for 10-days, 24 SAEs were detected, while in 1217 patients who did not receive remdesivir, 34 SAEs were observed. The control patients received a placebo in 2 trials and SOC in the other ones. A 10-day treatment with remdesivir was not associated with a significant change in the risk of SAEs due to AKI (RR = 0.71, 95% CI 0.43‒1.18, p = 0.19, with no detectable heterogeneity (I^2^ = 0.0%) (Fig. 3). The number of SAEs related to AKI in the Spinner study was 0 in both 5- and 10-day remdesivir regimens. Therefore, the results were not meta-analyzed with the serious AKI AEs reported in the Goldman study, to compare the risk between 5- and 10-day regimens of remdesivir.

### AKI classified as serious and non-serious AE

**Fig. 4:**
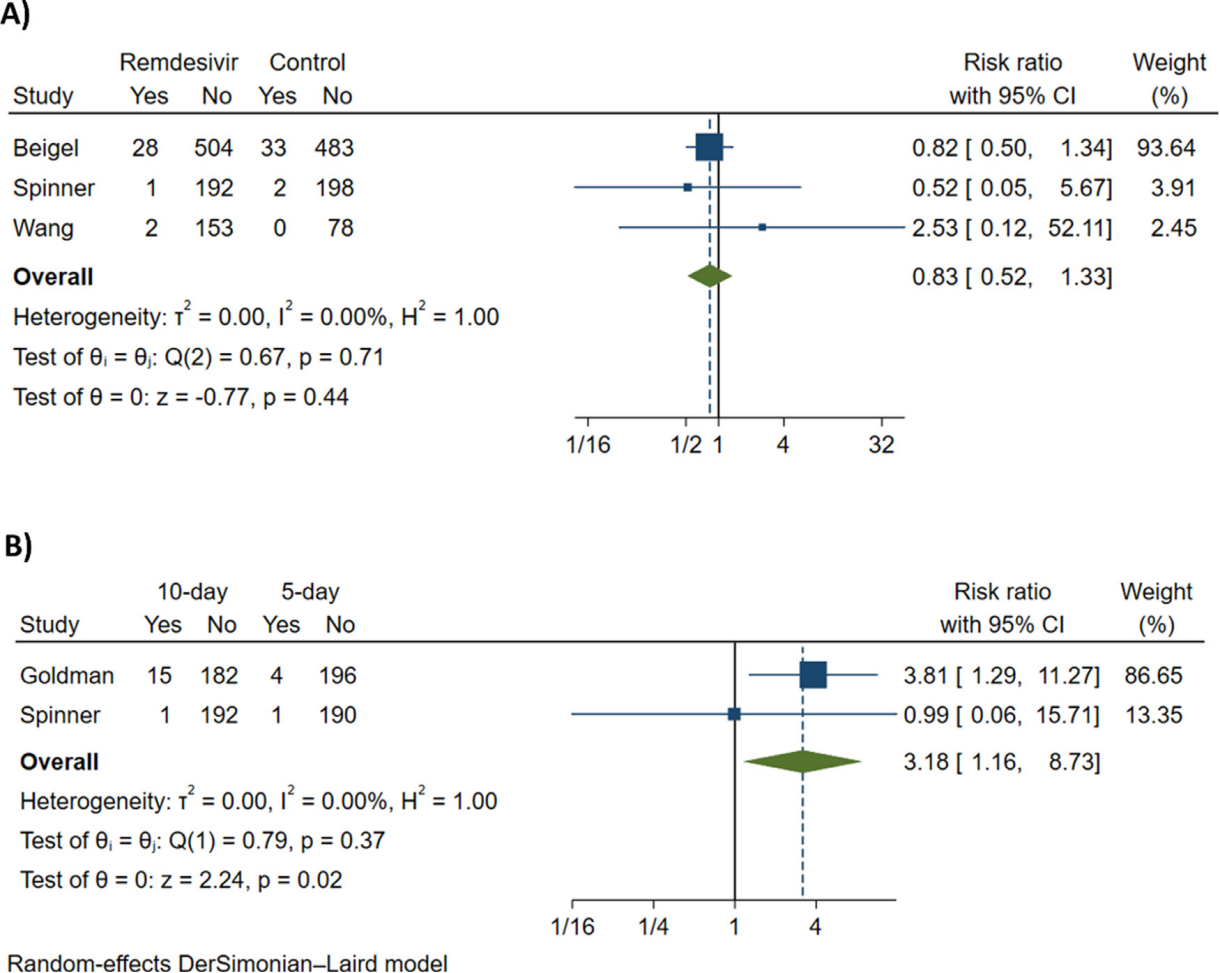
Forrest plot comparing the effect of remdesivir on AKI events classified as any grade of adverse event. (A) Comparing the effect of remdesivir vs control; (B) Comparing the effect of remdesivir treatment for 10 days vs. remdesivir treatment for 5 days.

Among the studies with a control group, 3 RCTs reported AKI events classified as both serious and non-serious AEs. In this regard, 880 patients received remdesivir for 10 days and 31 AEs of any grade due to AKI were reported. The number of combined serious and non-serious AEs due to AKI for the control patients was 35. The control patients received placebo in the Beigel and Wang studies and SOC in the Spinner study. The overall RR was 0.83 (95% CI 0.52‒1.33, p = 0.44), with no detectable heterogeneity (I^2^ = 0.0%) (Fig. 4A). However, comparing the number of AEs between 5- and 10-day treatment of remdesivir, there was a significant reduction in AEs related to AKI in 5-day remdesivir treatment versus 10-day remdesivir treatment (RR = 3.18, 95% CI 1.16‒8.73, p = 0.02), with no detectable heterogeneity (I^2^ = 0.0%) (Fig. 4B).

## Discussion

Remdesivir is an antiviral nucleotide analog that has been emergently approved for the treatment of hospitalized COVID-19 patients. Concerns about kidney safety of remdesivir have led to several studies focusing on AKI and other adverse renal events. AKI is characterized by a sudden decline in kidney function. Regarding the most recent and preferred AKI criteria suggested by the KDIGO 2012, the definition is based on rapid changes in serum creatinine level and urine output.^29^

In the current systematic review and meta-analysis of 2507 COVID-19 patients comparing remdesivir treatment for 10-days with the control group, remdesivir treatment was not accompanied by an increase in the serious or any grade AKI events, with low certainty of evidence. The AKI events reported were probably a complication of COVID-19 *per se*. Although the disease is generally recognized as a respiratory infection, it sometimes results in a multi-system disease that involves different organs of the body, including the kidney.^30^^,^^31^ There are several studies indicating that COVID-19 is associated with AKI, with an incidence ranging from 0.5% to 46%, depending on the COVID-19 severity as well as other baseline differences among study populations.^32^, ^33^, ^34^, ^35^, ^36^, ^37^, ^38^, ^39^, ^40^

Moreover, we meta-analyzed 2 RCTs with 781 COVID-19 patients and found very low certainty evidence that the risk of AKI events was significantly lower in patients receiving a 5-day regimen of remdesivir compared to 10-day treatment. However, in the Goldman study, the number of patients requiring invasive mechanical ventilation and high-flow oxygen support at baseline was higher in the 10-day remdesivir group compared to the 5-day group. As a result, patients were not balanced regarding the baseline disease severity and the higher number of AKI events may have been driven by the higher proportion of severely ill patients in the 10-day remdesivir group, given that severe COVID-19 is associated with a higher risk of AKI. Thus, further evidence is needed to delineate the effect of remdesivir or COVID-19 on AKI development.

This systematic review and meta-analysis had a comprehensive search strategy and included the latest published results of remdesivir RCTs. However, there are some limitations to the present study. First, few trials were included in this study which was due to the limited number of remdesivir RCTs that provided information on AKI events. Also, enough data was not available to perform subgroup analysis based on age and severity of the disease.

In conclusion, this study suggested that remdesivir probably does not increase the risk of AKI classified as serious or any grade adverse events. In turn, AKI events observed in COVID-19 patients treated with remdesivir may be mostly due to SARS-CoV-2 infection itself, dehydration/hypotension/shock, underlying diseases, and using well-known nephrotoxic medications such as nonsteroidal anti-inflammatory drugs or diuretics.

## Funding

This research did not receive any specific grant from funding agencies in the public, commercial, or not-for-profit sectors.

## CRediT authorship contribution statement

**Golnaz Shams:** Conceptualization, Investigation, Formal analysis, Methodology, Writing – original draft. **Asma Kazemi:** Investigation, Formal analysis, Methodology, Writing – review & editing. **Khatereh Jafaryan:** Investigation. **Mohammad Hossein Morowvat:** Resources, Writing – review & editing. **Payam Peymani:** Writing – review & editing. **Iman Karimzadeh:** Conceptualization, Writing – review & editing, Supervision.

## Conflicts of interest

The authors declare no conflicts of interest.

## Appendix Supplementary materials

Image, application 1

## Footnotes

Supplementary material associated with this article can be found, in the online version, at doi:10.1016/j.clinsp.2023.100200.
