# Supplementary material for: Acute kidney injury in COVID-19 patients receiving remdesivir: A systematic review and meta-analysis of randomized clinical trials
Source: Clinics (Sao Paulo). 2023 Apr 13;78:100200. doi: 10.1016/j.clinsp.2023.100200 (PMC10099148; doi:10.1016/j.clinsp.2023.100200)
Supplement: Supplementary file 1 [file mmc1.docx]

CLINICS-D-22-00572_Supplementary Material

**Search strategy**

Table S1 Search strategy in different databases.

| **PUBMED** (5/1/2022) | | |
| --- | --- | --- |
| **#1** | **remdesivir**  "remdesivir"[Supplementary Concept] OR "remdesivir"[All Fields] | 2,687 |
| **#2** | **(((((((((((((((((((((((((((coronavirus[Title/Abstract]) OR ("corona virus"[Title/Abstract])) OR (coronavirinae[Title/Abstract])) OR (coronaviridae[Title/Abstract])) OR (betacoronavirus[Title/Abstract])) OR (covid19[Title/Abstract])) OR ("covid 19"[Title/Abstract])) OR (nCoV[Title/Abstract])) OR ("CoV 2"[Title/Abstract])) OR (CoV2[Title/Abstract])) OR (sarscov2[Title/Abstract])) OR (SARS-CoV-2[Title/Abstract])) OR (2019nCoV[Title/Abstract])) OR ("novel CoV"[Title/Abstract])) OR ("wuhan virus"[Title/Abstract])) OR ((wuhan[Title/Abstract] OR hubei[Title/Abstract] OR huanan[Title/Abstract]) AND ( "severe acute respiratory"[Title/Abstract] OR pneumonia [Title/Abstract]) AND (outbreak[Title/Abstract])) ) OR (2019 novel coronavirus disease[Title/Abstract])) OR (COVID-19 pandemic[Title/Abstract])) OR (2019 novel coronavirus infection[Title/Abstract])) OR (2019-nCoV infection[Title/Abstract])) OR (coronavirus disease 2019[Title/Abstract])) OR (coronavirus disease-19[Title/Abstract])) OR (2019-nCoV disease[Title/Abstract])) OR (COVID-19 virus infection[Title/Abstract])) OR (covid-19[MeSH Terms])) OR (sars-cov-2[MeSH Terms])) OR (COVID-19[Supplementary Concept])) OR (severe acute respiratory syndrome coronavirus 2[Supplementary Concept])**  "coronavirus"[Title/Abstract] OR "corona virus"[Title/Abstract] OR "coronavirinae"[Title/Abstract] OR "coronaviridae"[Title/Abstract] OR "betacoronavirus"[Title/Abstract] OR "covid19"[Title/Abstract] OR "COVID-19"[Title/Abstract] OR "nCoV"[Title/Abstract] OR "CoV 2"[Title/Abstract] OR "CoV2"[Title/Abstract] OR "sarscov2"[Title/Abstract] OR "SARS-CoV-2"[Title/Abstract] OR "2019nCoV"[Title/Abstract] OR "novel CoV"[Title/Abstract] OR "wuhan virus"[Title/Abstract] OR (("wuhan"[Title/Abstract] OR "hubei"[Title/Abstract] OR "huanan"[Title/Abstract]) AND ("severe acute respiratory"[Title/Abstract] OR "pneumonia"[Title/Abstract]) AND "outbreak"[Title/Abstract]) OR "2019 novel coronavirus disease"[Title/Abstract] OR "covid 19 pandemic"[Title/Abstract] OR "2019 novel coronavirus infection"[Title/Abstract] OR "2019 ncov infection"[Title/Abstract] OR "coronavirus disease 2019"[Title/Abstract] OR "coronavirus disease 19"[Title/Abstract] OR "2019 ncov disease"[Title/Abstract] OR "covid 19 virus infection"[Title/Abstract] OR "COVID-19"[MeSH Terms] OR "SARS-CoV-2"[MeSH Terms] OR "COVID-19"[Supplementary Concept] OR "severe acute respiratory syndrome coronavirus 2"[Supplementary Concept] | 260,708 |
| **#3** | **(((((((((((randomized controlled trial[Publication Type]) OR (controlled clinical trial[Publication Type])) OR (randomized[Title/Abstract])) OR (randomly[Title/Abstract])) OR (trial[Title/Abstract])) OR (placebo[Title/Abstract])) OR (groups[Title/Abstract])) OR (drug therapy[Title/Abstract]) ) OR (clinical trials as topic[MeSH Terms])) OR (controlled clinical trials as topic[MeSH Terms])) OR (controlled clinical trials, randomized[MeSH Terms])) OR (randomized controlled trials as topic[MeSH Terms])**  "randomized controlled trial"[Publication Type] OR "controlled clinical trial"[Publication Type] OR "randomized"[Title/Abstract] OR "randomly"[Title/Abstract] OR "trial"[Title/Abstract] OR "placebo"[Title/Abstract] OR "groups"[Title/Abstract] OR "drug therapy"[Title/Abstract] OR "clinical trials as topic"[MeSH Terms] OR "controlled clinical trials as topic"[MeSH Terms] OR "randomized controlled trials as topic"[MeSH Terms] OR "randomized controlled trials as topic"[MeSH Terms] | 3,689,700 |
| #4 | #1 AND #2 AND #3 | 615 |

| **Cochrane CENTRAL** (5/1/2022) | | |
| --- | --- | --- |
| #1 | Remdesivir | 320 |
| #2 | (2019 novel coronavirus disease):ti,ab,kw | 417 |
| #3 | COVID19 | 607 |
| #4 | COVID 19 | 10411 |
| #5 | COVID-19 | 10380 |
| #6 | SARS-CoV-2 | 441 |
| #7 | coronavirus 2019 | 4036 |
| #8 | COVID-19 virus disease | 1482 |
| #9 | 2019 novel coronavirus infection | 265 |
| #10 | coronavirus disease 2019 | 3895 |
| #11 | coronavirus disease-19 | 85 |
| #12 | COVID-19 virus infection | 1296 |
| #13 | #2 OR #3 OR #4 OR #5 OR #6 OR #7 OR #8 OR #9 OR #10 OR #11 OR #12 | 10781 |
| #14 | #1 AND #13 | 310 |

| **Web of Science** (5/1/2022) | | |
| --- | --- | --- |
| **#1** | **TS=(remdesivir)** | 2,356 |
| **#2** | **((((((((((((((TS=(coronavirus)) OR TS=(covid)) OR TS=(Covid19)) OR TS=(ncov)) OR TS=((SARS**  **NEAR/3 COV))) OR TI=("novel coron*virus")) OR TI=(2019*ncoV)) OR**  **TS=(2019ncov)) OR TS=((CORON*VIRUS NEAR/3 (OUTBREAK OR pandemic**  **OR 2019 OR new OR novel)))) OR TS=(coronavirinae)) OR TS=(coronaviridae)) OR**  **TS=(betacoronavirus)) OR TS=(SarS2)) OR TS=(COV2)) OR TS=(”corona pandemic”)** | 306,193 |
| **#3** | **TS=((((((clinical near trial* or controlled near trial* or crossover or “cross over”) or ((single* or doubl* or trebl* or tripl*) near (blind* or mask*)) or (singleblind* or doubleblind* or trebleblind* or tripleblind* or placebo* or random*))))))** | 2,865,536 |
| #4 | #1 AND #2 AND #3 | 642 |

| **bioRxiv and medRxiv** (5/1/2022) | | |
| --- | --- | --- |
| #1 | (Covid OR coV2 OR coronavirus OR “sars cov” OR ncov) AND (remdesivir) | 1047 |

| **Scopus** (2/15/2022) | | |
| --- | --- | --- |
| #1 | TITLE-ABS-KEY(coronavir* OR "corona virus" OR "corona pandemic" OR betacoronavir* OR covid19 OR covid OR ncov OR "CoV 2" OR coV2 OR sarscoV2 OR sarS2 OR 2019ncov OR "novel CoV" OR "wuhan virus") OR TITLE-ABS-KEY(sars AND cov) OR TITLE-ABS-KEY(wuhan OR hubei OR huanan) OR TITLE-ABS-KEY( "severe acute respiratory" OR pneumonia*) OR TITLE-ABS-KEY(outbreak*) AND TITLE-ABS-KEY(remdesivir) AND (INDEXTERMS( "clinical trial" OR "controlled trial" OR "random allocation" OR "Double-Blind" OR "Single-Blind" OR "Cross-Over" OR "crossover" OR "multicenter study" OR "randomization" OR "controlled study" OR "placebo" ) OR TITLE-ABS-KEY ( "clinical trial" OR "controlled trial" OR "random allocation" OR "randomize" OR "randomise" OR "randomly allocated" OR "allocated randomly" OR "Double-Blind" OR "Single-Blind" OR "Cross-Over" OR "crossover" OR "Placebo" ) OR TITLE-ABS ( clinical trial* OR trial* OR rct* OR random* OR blind* )) | 2,911 |

| Table S2 Certainty of evidence assessment | | | | | | | | | | | | | |
| --- | --- | --- | --- | --- | --- | --- | --- | --- | --- | --- | --- | --- | --- |
| **Certainty assessment** | | | | | | | **№ of patients** | | **Effect** | | | **Certainty** | **Importance** |
| **№ of studies** | **Study design** | **Risk of bias** | **Inconsistency** | **Indirectness** | **Imprecision** | **Other considerations** | **Remdesivir** | **placebo/standard of care** | **Relative (95% CI)** | **Absolute (95% CI)** |  | |  |
| **Serious adverse event** | | | | | | | | | | | | | |
| 4 | randomised trials | serious^a^ | not serious | not serious | serious^b^ | none |  |  | **RR 0.71** (0.43 to 1.18) | **1 fewer per 1,000** (from 1 fewer to 0 fewer) | ⨁⨁◯◯ Low | | CRITICAL |
| **Any adverse events of AKI** | | | | | | | | | | | | | |
| 4 | randomised trials | serious^a^ | not serious | not serious | serious^b^ | none |  |  | **RR 0.83** (0.52 to 1.33) | **1 fewer per 1,000** (from 1 fewer to 1 fewer) | ⨁⨁◯◯ Low | | CRITICAL |
| **Effect of remdesivir 10-day course vs remdesivir 5-day course on any AKI** | | | | | | | | | | | | | |
| 2 | randomised trials | very serious^c^ | not serious | not serious | serious^b^ | none |  |  | **RR 3.18** (1.16 to 8.73) | **3 fewer per 1,000** (from 9 fewer to 1 fewer) | ⨁◯◯◯ Very low | |  |

**CI:** confidence interval; **RR:** risk ratio

#### Explanations

a. Downgraded by one level since >50% of studies were at some concern for risk of bias

b. downgraded by one level for imprecision since 95% CI includes important benefit/harm (SRR <0.75 and/or >1.25(

c. Both two studies were at high risk of bias or some concern


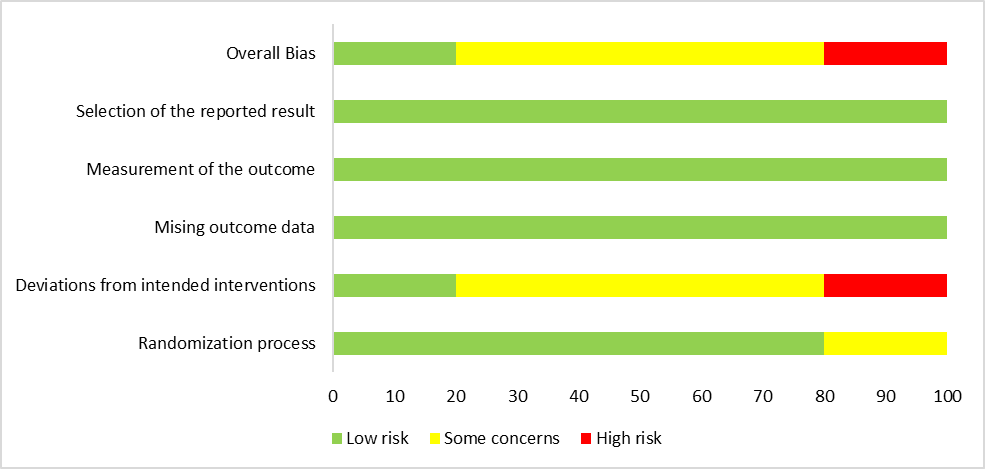


Figure S1. Risk of bias graph.
